# Changes in patient health questionnaire (PHQ-9) scores in adults with medical authorization for cannabis

**DOI:** 10.1186/s12889-020-09089-3

**Published:** 2020-06-23

**Authors:** Jessica M. Round, Cerina Lee, John G. Hanlon, Elaine Hyshka, Jason R. B. Dyck, Dean T. Eurich

**Affiliations:** 1 School of Public Health, University of Alberta, Edmonton, Alberta Canada; 2 St. Michael’s Hospital Department of Anesthesia, University of Toronto, Toronto, Ontario Canada; 3 Department of Anesthesia and Pain Medicine, University of Toronto, Toronto, Ontario Canada; 4 Cardiovascular Research Centre, Department of Pediatrics, Faculty of Medicine and Dentistry, University of Alberta, Edmonton, Alberta Canada

**Keywords:** Depression, Major depressive disorder, Patient health questionnaire, PHQ-9, Medical cannabis

## Abstract

**Background:**

Legal access to medical cannabis is increasing world-wide. Despite this, there is a lack of evidence surrounding its efficacy on mental health outcomes, particularly, on depression. This study assesses the effect of medical cannabis on Patient Health Questionnaire (PHQ-9) scores in adult patients between 2014 and 2019 in Ontario and Alberta, Canada.

**Methods:**

An observational cohort study of medically authorized cannabis patients in Ontario and Alberta. Overall change in PHQ-9 scores from baseline to follow-up were evaluated (mean change) over a time period of up to 3.2 years.

**Results:**

37,338 patients from the cohort had an initial PHQ-9 score recorded with 5103 (13.7%) patients having follow-up PHQ-9 scores. The average age was 54 yrs. (SD 15.7), 46% male, 50% noted depression at baseline. The average PHQ-9 score at baseline was 10.5 (SD 6.9), following a median follow-up time of 196 days (IQR: 77–451) the average final PHQ-9 score was 10.3 (SD 6.8) with a mean change of − 0.20 (95% CI: − 0.26, − 0.14, *p-*value < 0.0001). Overall, 4855 (95.1%) had no clinically significant change in their PHQ-9 score following medical cannabis use while 172 (3.4%) reported improvement and 76 (1.5%) reported worsening of their depression symptoms.

**Conclusions:**

Although the majority showed no clinically important changes in PHQ-9 scores, a number of patients showed improvement or deteriorations in PHQ-9 scores. Future studies should focus on the parallel use of screening questionnaires to control for PHQ-9 sensitivity and to explore potential factors that may have attributed to the improvement in scores pre- and post- 3-6 month time period.

## Background

The medical use of cannabis has become a world-wide phenomenon – with increasing numbers of jurisdictions allowing patient access to cannabis for a variety of therapeutic interventions [1]. Canadians have had legal access to medical cannabis [2] for its treatment of a variety of health conditions [3], including for the improvement of mental health outcomes [4–6]. Despite its availability, significant evidence gaps remain, particularly for depression and depression-related health outcomes [7–11]. Indeed, there is a lack of rigorous large-patient cohort studies on medical cannabis that utilize standardized validation tools on determining its impact on mental health [12, 13].

Pre-existing clinical studies and systematic reviews on medical cannabis’ impact on depression and depression-related outcomes show mixed results. To date, the most recent clinical recommendations from both Canada and the US (based on the best-available evidence) [14, 15] report that there is limited evidence on cannabis’ efficacy in improving depression symptoms. Importantly, few studies have directly studied the effect of medical cannabis solely on depression [16–19]. Rather, the majority of studies categorize depression under the broad category of mental health outcomes [5, 20, 21]. Furthermore, the studies on depression are themselves, limited, as very few utilize the Patient Health Questionnaire (PHQ-9) [22] as a gold standard for measuring depression outcomes [19, 23, 24]. Likewise, these studies are predominantly designed with small cohort sample sizes [25], focus on how cannabis consumption may cause/develop depression [26, 27] - rather than improve it [13, 28]; very few differentiate medical cannabis use from nonmedical use [29]; and lastly, studies frequently emphasize the limitations of inferences made between medical cannabis and depression due to contemporaneous use of other drugs or illegal substances amongst participants [13, 30]. Thus, this study was designed to provide clarity of the potential impact of medical cannabis on depression and depression-related health outcomes by measuring changes in patients’ PHQ-9 scores over time.

## Methods

### Study design

Cohort study of patients in Alberta and Ontario, Canada who were authorized medical cannabis between 2014 and 2019.

### Study population

#### Inclusion criteria

The study population consisted of all adult patients authorized to access medical cannabis attending a chain of specialized clinics in the provinces of Alberta and Ontario (Canada). Participants were adults of any sex, ethnicity, and socioeconomic status who were seeking medical cannabis for any reason. Patients may choose to seek assessment for medical cannabis through the clinic via a self-referral or by a physician referral.

#### Exclusion criteria

All patients without a PHQ-9 questionnaire at baseline (i.e., at time of medical cannabis authorization), and those without at least one score from any point during the follow-up period were excluded as we were most interested in the changes in the PHQ-9 over time.

### Data source

Informed signed consent was provided by the patient at the time of first referral which allowed data to be collected and used for clinical and research purposes. All data was released as de-identified data to the researchers. The self-reported outcomes and physician-based medical assessments were collected from adult patients at cannabis clinics in Alberta and Ontario, Canada who have been authorized to use medical cannabis. As part of the intake process, each patient seeking medical cannabis meets with a counselor who performs an initial assessment and collects relevant data. All patients must provide sociodemographic information and disclose their primary medical complaints that constitute their rationale for requesting a medical cannabis authorization. In addition, patients completed several validated questionnaires at baseline, including: pain questionnaires [31, 32], the Generalized Anxiety Disorder 7-item (GAD-7) scale [33]; Patient Health Questionnaire (PHQ-9) [22]; and the CAGE Questionnaire Adapted to Include Drugs (CAGE-AID) [34], among others. Informed consent is provided by the patient at the time of first referral, which allows data to be collected and used by the clinics. Following their initial intake interview, the patient is referred to a physician who makes their assessment based on the self-reported information as well as the patient’s health record. All data was released as de-identified data. 

### Patient and public involvement

Patients and the public were not involved in the design, conduct and reporting of this research project as it was not applicable to this project.

### Ethics approval

This study was approved by the University of Alberta Health Research Ethics board (PRO 00068887) and Veritas Research Ethics Board in Ontario (16111–13:21:103–01-2017). Informed signed consent is provided by the patient at the time of first referral, which allows data to be collected and used by Canadian Cannabis Clinics.

### Outcomes

The PHQ-9 questionnaire is a self-administered tool for assessing depressive disorders [22] using nine items scored on their frequency from ‘not at all’ to ‘nearly every day’ resulting in a score between 0 and 27 (higher scores representing greater depressive symptoms). This method for assessing depression symptoms was chosen because many of the nine items align with the DSM-V criteria for identifying depressive disorders. The questionnaire is also straightforward and is often used clinically as the measure can be rapidly completed by patients to effectively assess depression symptoms [22]. The PHQ-9 has a reported sensitivity of 88% and a specificity of 88% for major depression. One major limitation of the PHQ-9 is that it only screens for depression and depression-related symptoms; it cannot diagnose Major Depressive Disorder or other depressive-like disorders. The justification for the use of the PHQ-9 was simply due to the fact that it was the main selected tool utilized by the physicians at the cannabis clinics to assess and screen for depression.

The PHQ-9 scores were also used to categorize the severity of the depression symptoms: score of 0–4 was no symptoms, 5–9 mild, 10–14 moderate, 15–19 moderately severe, and 19–27 severe symptoms. These are the depression categories that are recommended for use with the PHQ-9 questionnaire [22].

Covariates including age, sex, neighbourhood average income quintile, length of follow-up, reason for cannabis use, method of cannabis use, and antidepressant usage were considered. Neighbourhood average income was determined using census data matched to the patient’s area of residence. Length of follow-up was the number of days between the initial administration of the PHQ-9 and the follow-up administration of the PHQ-9. Reason for cannabis use was coded into the following categories: pain, mental health, autoimmune disorder, cancer, sleep problems, neurological disorder, gastrointestinal disorder, and other. The mental health category was further broken down to consider anxiety, depression, post-traumatic stress disorder, bipolar disorder, panic disorder, and mood disorder. See Additional file 1 for the phrases used for coding the reasons for visit. Some patients did not record a reason for seeking medical cannabis so these patients were coded as having an unknown reason for use. Method of cannabis use was coded into ingesting, smoking, vaping, or topical use (see Additional file 1 for keywords used). Antidepressant usage was coded into the following categories: selective serotonin reuptake inhibitors (SSRI), serotonin-norepinephrine reuptake inhibitors (SNRI), tricyclic antidepressants (TCA), and other antidepressants. Other antidepressants included norepinephrine–dopamine reuptake inhibitors (NDRI), noradnergic and specific serotonergic antidepressants (NaSSA), and Monoamine oxidase inhibitors (MAOI). Patients were considered to have used antidepressants if any of these classes of medications were listed as current medications at any appointment during the patient’s care at the cannabis clinics(see Additional file 1 for a full list of medications included in each antidepressant category). Information on the frequency of use and dosage was not available. Both the method of cannabis use and antidepressant categories were not mutually exclusive as many patients use multiple methods to consume cannabis and multiple classes of antidepressants.

### Statistical analysis

Descriptive statistics using counts, percentages, means, and standard deviation were used to assess the patients’ demographics. The changes in PHQ-9 scores and depression categories were determined by subtracting the final PHQ-9 score from the initial PHQ-9 score. Therefore, a negative change in PHQ-9 represents a lessening of depression symptoms while a positive change in PHQ-9 represents an increase in depression symptoms. Any follow-up appointment where depression symptoms were reassessed was considered for analysis, therefore some subjects had multiple follow-up PHQ-9 scores for multiple follow-up appointments. The follow-up scores with the longest length of follow-up were used to assess changes in PHQ-9 scores and depression categorization. For the purpose of this study, we considered a 5-point change in PHQ-9 to be potentially clinically significant, as others have [35]. To examine the differences between the initial and final PHQ-9 scores and depression categories paired t-tests were conducted. A multiple linear regression was also conducted to assess the effects of covariates on their PHQ-9 scores. In addition, as the PHQ-9 is scored between 0 and 27, in instances where the recorded PHQ-9 score was outside of the plausible range, these scores were omitted from analysis. Statistical analysis was conducted using Stata version 15.1.

### Sensitivity analysis

Follow-up time was broken in 6 categories to assess if length of exposure to cannabis had an effect on PHQ-9 scores. The time categories were as follows: less than 7 days, 1 week to 3 months, 3 to 6 months, 6 to 12 months, 1 to 2 years, and greater than 3 years. Post-hoc, we looked specifically at patients who reported depression at onset as well as patients who had at least 6 months of follow-up data to evaluate the change in PHQ-9 scores among these specific groups as clinically changes in depression generally do not occur rapidly in patients.

## Results

**Fig. 1:**
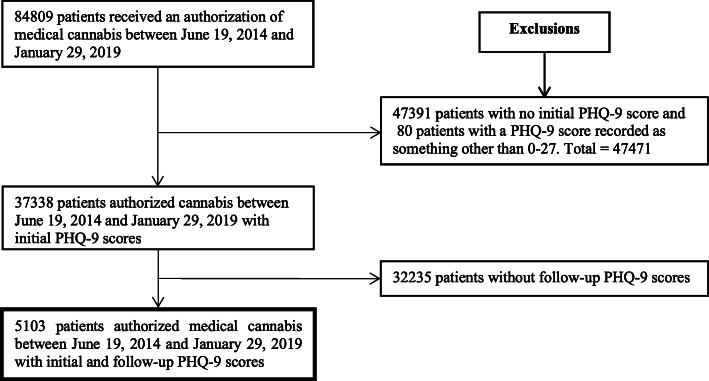
Selection of Study Population

84,809 patients were authorized medical cannabis with 37,338 (44%) completing the PHQ-9 questionnaire at baseline and 5103 (6.0%) patients during the follow-up (Fig. 1). Multiple follow-up appointments occurred for some patients resulting in 5795 follow-up PHQ-9 scores from the 5103 patients.

Importantly, the characteristics of those with and without follow-up PHQ-9 scores were overall similar (Table 1) and the same distribution of depression categories existed. The reasons for seeking medical cannabis were also similar among those who did and did not have follow-up PHQ-9 scores with the top reasons being pain, autoimmune disorders, sleep problems, and mental health disorders. Among those seeking medical cannabis for mental health disorders (*N* = 12,883), 41.0% self-identified as having depression. 17,491 (47%) patients who completed the PHQ-9 questionnaire at baseline were using antidepressants to manage their depression symptoms while attending the cannabis clinics. The timing of follow-up appointments varied: 10.6% (less than 7 days), 26.0% (1 week to ≤ 3 months), 18.2% (3 to ≤ 6 months), 18.4% (6 to 12 months), 20.6% (1 to 2 years), and 6.2% (greater than 2 years) (Table 2). The median (IQR) time for all follow-up appointments was 196 days (77–451).

**Table 1:** Characteristics of Patients Authorized Medical Cannabis Patients and Screened Using the PHQ-9 Questionnaire (*n* = 37,338)

Characteristic	All Patients with PHQ-9 Scores (*N* = 37,338)	Patients with PHQ-9 Follow-up (*N* = 5103)	Patients without PHQ-9 Follow-up (*N* = 32,235)
	N (%)		
**Age** (years)			
< 21	210 (0.6)	21 (0.4)	189 (0.6)
21–30	2504 (6.7)	277 (5.4)	2227 (6.9)
31–40	5641 (15.1)	847 (16.6)	4794 (14.9)
41–50	6637 (17.8)	990 (19.4)	5647 (17.5)
51–60	9143 (24.5)	1342 (26.3)	7801 (24.2)
61–70	7389 (19.8)	996 (19.5)	6393 (19.8)
71–80	3956 (10.6)	437 (8.6)	3519 (10.9)
81–90	1637 (4.4)	172 (3.4)	1465 (4.5)
> 90	221 (0.6)	21 (0.4)	200 (0.6)
**Sex**			
Female	20,144 (53.9)	2811 (55.1)	17,333 (53.8)
Male	17,193 (46.1)	2292 (44.9)	14,901 (46.2)
Other	1 (0.0)	–	1 (0.0)
**Neighbourhood Income Quintile**			
1	7168 (19.2)	1044 (20.5)	6124 (19.0)
2	7781 (20.8)	913 (17.9)	6868 (21.3)
3	7912 (21.3)	1017 (20.0)	6895 (21.5)
4	8613 (23.1)	1270 (24.9)	7343 (22.8)
5	5673 (15.2)	832 (16.3)	4841 (15.0)
**PHQ-9 Score,** Mean (SD)	10.45(6.88)	10.48(6.83)	10.44(6.88)
**Depression Category**			
None	8777 (23.5)	1181 (23.1)	7596 (23.6)
Mild	9769 (26.2)	1372 (26.9)	8397 (26.1)
Moderate	8005 (21.4)	1069 (21.0)	6936 (21.5)
Moderately Severe	6106 (16.4)	833 (16.3)	5273 (16.4)
Severe	4681 (12.5)	648 (12.7)	4033 (12.5)
**Reason for Cannabis Use**			
Pain	29,537(79.1)	4230 (82.9)	25,307 (78.5)
Mental Health	14,884 (39.9)	1955 (38.3)	12,929 (40.1)
Anxiety	9315 (25.0)	1242 (24.3)	8073 (25.0)
Depression	6100 (16.3)	785 (15.4)	5315 (16.5)
Post-traumatic Stress Disorder	1478 (4.0)	181 (3.6)	1297 (4.0)
Bipolar	773 (2.1)	82 (1.6)	691 (2.1)
Panic Disorder	1340 (4.0)	160 (3.1)	1180 (3.7)
Mood Disorder	1448 (3.9)	171 (3.4)	1277 (4.0)
Autoimmune	8481 (22.7)	1281 (25.1)	7200 (22.3)
Cancer	3860 (10.3)	416 (8.1)	3444 (10.7)
Sleep problems	9064 (24.3)	1174 (23.0)	7890 (24.5)
Neurological	3005 (8.1)	403 (7.9)	2602 (8.1)
Gastrointestinal	1774 (4.8)	283 (5.6)	1491 (4.6)
Other	6268 (16.8)	859 (16.8)	5409 (16.8)
Uncategorized	2450 (6.6)	227 (4.5)	2223 (6.9)
**Method of Cannabis Use**			
Ingesting	22,021 (59.0)	4016 (78.7)	18,005 (55.9)
Smoking	12,719 (34.1)	2491 (48.8)	10,228 (31.7)
Vaping	13,745 (36.8)	2771 (54.3)	10,994 (34.1)
Topical	324 (0.9)	41 (0.8)	283 (0.9)
**Antidepressant Usage**	17,491 (46.8)	2656 (52.0)	14,835 (46.0)
SSRI	7647 (20.5)	1100 (21.6)	6547 (20.3)
SNRI	8056 (21.6)	1279 (25.1)	6777 (21.0)
TCA	3621 (9.7)	602 (12.1)	3019 (9.4)
Other	2974 (8.0)	463 (9.1)	2511 (7.8)

*SSRI* selective serotonin reuptake inhibitor, *SNRI* serotonin-norepinephrine reuptake inhibitor, *TCA* tricyclic antidepressants

**Table 2:** Timing of all follow-up appointments (*n* = 5795) with the mean difference in PHQ-9 scores for each time period

Follow-up Appointment Time	N (%)	Mean Difference in PHQ-9 Score (95% CI)	***p***-value
≤7 days	614 (10.6)	0.04 (−0.09, 0.17)	0.547
1 week-3 months	1509 (26.0)	− 0.25 (− 0.36, − 0.14)	< 0.0001
3–6 months	1056 (18.2)	− 0.47 (− 0.67, − 0.27)	< 0.0001
6–12 months	1066 (18.4)	− 0.28 (− 0.41, − 0.14)	0.0001
1–2 years	1193 (20.6)	−0.15 (− 0.29, − 0.01)	0.038
> 2 years	357 (6.2)	−0.24 (− 0.52, 0.04)	0.093

The average PHQ-9 score at baseline was 10.5 (SD 6.9). Following an average follow-up time of 255 days (SD: 250) (median: 196, IQR: 77–451), the average final PHQ-9 score was 10.3 (SD 6.8) with a mean change of − 0.20 (95% CI: − 0.26, − 0.14, *p-*value < 0.0001). Overall, most patients had minimal change in their PHQ-9 score (88.3%) or depression categorization (92.7%) (Table 3 & Additional file 2). However, of the 5103 followed-up patients, 172 (3.4%) had what would be considered a clinically important decrease of at least 5 points or more and 76 (1.5%) had an increase following medical cannabis use.

**Table 3:** Changes in depression classification from initial to final appointment based on initial depression categorization (*n* = 5103)

Depression Category Change	N (%)	Mean Difference in PHQ-9 Score (95% CI)
**Severe (*n*** **= 648)**		−0.70 (− 0.94, − 0.46)
Severe to None	8 (1.2)	
Severe to Mild	9 (1.4)	
Severe to Moderate	3 (0.5)	
Severe to Moderately Severe	20 (3.1)	
No Change	608 (93.8)	
**Moderately Severe (*n*** **= 833)**		−0.44 (−0.61, −0.27)
Moderately Severe to None	9 (1.1)	
Moderately Severe to Mild	20 (2.4)	
Moderately Severe to Moderate	21 (2.5)	
Moderately Severe to Severe	14 (1.7)	
No Change	769 (92.3)	
**Moderate (*n*** **= 1069)**		−0.41 (−0.54, −0.28)
Moderate to None	38 (3.6)	
Moderate to Mild	37 (3.5)	
Moderate to Moderately Severe	16 (1.5)	
Moderate to Severe	3 (0.3)	
No Change	975 (91.2)	
**Mild (*n*** **= 1372)**		−0.04 (−0.13, 0.05)
Mild to None	72 (5.3)	
Mild to Moderate	35 (2.6)	
Mild to Moderately Severe	10 (0.7)	
Mild to Severe	1 (0.1)	
No Change	1254 (91.4)	
**None (*n*** **= 1181)**		0.25 (0.14, 0.35)
None to Mild	44 (3.7)	
None to Moderate	12 (1.0)	
None to Moderately Severe	1 (0.1)	
None to Severe	2 (0.2)	
No Change	1122 (95.0)	

Subsequent analysis, based on patient’s initial depression classification showed similar results (Table 3 & Additional file 2), although the clinical importance of these changes is uncertain. For patients with severe depression (*n =* 648), 5.1% had a decrease of at least five points in their PHQ-9 score, the mean difference from initial to final appointment was − 0.70 (95% CI: − 0.94, − 0.46, *p-*value < 0.0001). For patients with moderately severe depression (*N =* 833), 4.9% had a decrease and 0.6% had an increase of at least five points in their PHQ-9 score, the mean difference was − 0.44 (95% CI: − 0.61, − 0.27, *p-*value < 0.0001). Conversely, for patients with no depression (*N =* 1181), 2.5% had an increase in their PHQ-9 score, the mean difference was 0.25 (95% CI: 0.14, 0.35, *p-*value < 0.0001).

The multiple linear regression analyses showed that initial PHQ-9 score, the presence of pain, mental health disorders, and depression at baseline, and the use of SSRIs were statistically associated with some change in PHQ-9 scores (Additional file 3). None of the individual coefficients contributed to a change in PHQ-9 score of at least five points suggesting that while they are statistically significant, they may not be clinically important.

### Sensitivity analyses results

**Fig. 2:**
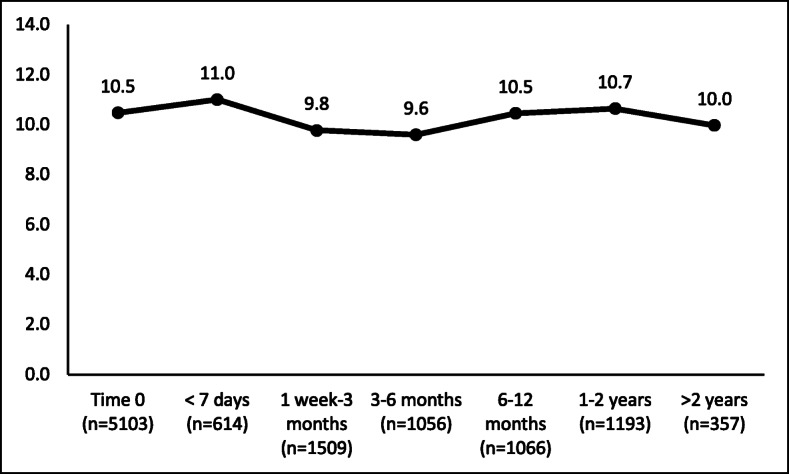
Mean PHQ-9 Scores Over Time

The follow-up period that had the most frequent changes in the depression categorization was 3 to 6 months following authorization (Fig. 2 and Table 4). Of the 1056 patients (20.7%) whose PHQ-9 score was re-assessed three to six-months following authorization, pronounced changes in scores included: 11.1% had a decrease and 5.6% had an increase in their depression categorization (Table 4). The mean difference in PHQ-9 score for this time period was − 0.47 (95% CI: − 0.67, − 0.27, *p-*value< 0.0001) (Table 2). While the majority of patients had no change in their depression categorization, for those that did experience a significant numeric change in score- there were roughly twice as many patients with a decrease in depression categorization than an increase, with the exception of the less than 7 days time period.

**Table 4:** Change in depression categories for each patient’s follow up PHQ-9 testing over each patient’s entire follow-up period. (*n* = 5795)

	All Appointment	< 7 days	1 week-3 months	3–6 months	6–12 months	1–2 years	> 2 years
	(*n* = 5795)	(*n* = 614)	(*n* = 1509)	(*n* = 1056)	(*n* = 1066)	(*n* = 1193)	(*n* = 357)
**Decreased Depression**	330 (5.7)	9 (1.5)	93 (6.2)	117 (11.1)	44 (4.3)	46 (3.9)	21 (5.9)
Severe to None	11 (0.2)	–	–	3 (0.3)	3 (0.3)	3 (0.3)	2 (0.6)
Severe to Mild	11 (0.2)	1 (0.2)	2 (0.1)	2 (0.2)	5 (0.5)	1 (0.1)	–
Severe to Moderate	5 (0.1)	–	–	2 (0.2)	2 (0.2)	1 (0.1)	–
Severe to Moderately Severe	20 (0.4)	1 (0.2)	4 (0.3)	8 (0.8)	1 (0.3)	6 (0.5)	–
Moderately Severe to None	15 (0.3)	–	4 (0.3)	5 (0.5)	3 (0.3)	3 (0.3)	–
Moderately Severe to Mild	25 (0.4)	–	6 (0.4)	8 (0.8)	5 (0.5)	4 (0.3)	2 (0.6)
Moderately Severe to Moderate	26 (0.4)	1 (0.2)	5 (0.3)	7 (0.7)	6 (0.6)	4 (0.3)	3 (0.8)
Moderate to None	53 (0.9)	3 (0.5)	16 (1.1)	16 (1.5)	5 (0.5)	9 (0.8)	4 (1.1)
Moderate to Mild	61 (1.1)	1 (0.2)	23 (1.5)	21 (2.0)	9 (0.8)	4 (0.3)	3 (0.8)
Mild to None	103 (1.8)	2 (0.3)	33 (2.2)	45 (4.3)	5 (0.5)	11 (0.9)	7 (2.0)
**Increased Depression**	188 (3.3)	17 (2.8)	48 (3.2)	59 (5.6)	27 (2.2)	25 (2.1)	12 (3.4)
None to Severe	5 (0.1)	–	–	2 (0.2)	–	3 (0.3)	–
Mild to Severe	3 (0.1)	–	–	2 (0.2)	–	–	1 (0.3)
Moderate to Severe	5 (0.1)	–	1 (0.1)	1 (0.1)	2 (0.2)	–	1 (0.3)
Moderately Severe to Severe	17 (0.3)	1 (0.2)	5 (0.3)	4 (0.4)	3 (0.3)	4 (0.3)	–
None to Moderately Severe	1 (0.02)	–	–	1 (0.1)	–	–	–
Mild to Moderately Severe	13 (0.2)	1 (0.2)	1 (0.1)	5 (0.5)	–	4 (0.3)	2 (0.5)
Moderate to Moderately Severe	21 (0.4)	4 (0.7)	7 (0.5)	2 (0.2)	6 (0.6)	–	2 (0.6)
None to Moderate	19 (0.3)	2 (0.3)	5 (0.3)	5 (0.5)	2 (0.2)	5 (0.4)	
Mild to Moderate	44 (0.8)	6 (1.0)	12 (0.8)	14 (1.3)	5 (0.5)	5 (0.4)	2 (0.6)
None to Mild	60 (1.0)	3 (0.5)	17 (1.1)	23 (2.2)	9 (0.8)	4 (0.3)	4 (1.1)
**No Change**	5277 (91.1)	588 (95.8)	1368 (90.7)	880 (83.3)	995 (93.3)	1122 (94.1)	324 (90.8)

In our post-hoc analysis, medical cannabis authorization was not associated with a clinically important change in the PHQ-9 scores among those with initial depression classification of moderate to severe. Among these 2550 patients, the mean change in PHQ-9 score was − 0.49 (95% CI: − 0.59, − 0.39, *p-*value< 0.0001). When restricting the analysis to patients with a follow-up of greater than 6 months (*n =* 2616) there remained no clinically important change in PHQ-9, mean change in PHQ-9 score: -0.30 (95% CI: − 0.39, − 0.20, *p-*value< 0.0001).

## Discussion

This population-based study of patients in Ontario and Alberta authorized for medical cannabis showed some clinical impact on depression symptoms as measured by the PHQ-9, in particular, 20.7% (1056) of patients - whose PHQ-9 score was re-assessed 3 to 6-months following authorization – reported a significant change in scores. However, at the population level, the overall majority of patients experienced minimal detriments to their mental health, which is reassuring for clinicians and patients using medical cannabis. Moreover, there is a subset of patients that improved over time, although it is uncertain how clinically important this improvement is as overall effects were relatively small. Many of the patients were using antidepressants while also using medical cannabis, however SSRIs were the only class of antidepressant statistically associated with a change in PHQ-9 scores, however the clinical importance of this interaction remains unknown. Given wide variability of the type of cannabis products or cannabis cultivars used, we would expect that any PHQ-9 differences would be hard to identify, however based on this study we found that the method of cannabis use does not significantly predict a change in PHQ-9 score. Despite this variability, subgroups of individuals were identified for scores that showed both improvement and worsening of symptoms.

Current recommendations from Canadian clinicians [14] and The National Academies of Science, Engineering, and Medicine [15] concur that there is very limited to insufficient evidence for medical cannabis’ efficacy in improving depression-related outcomes. From the literature, previous studies [16–19, 29] specifically utilizing the PHQ-9 tool to measure depression levels in association with (medicinal or recreational) cannabis use have shown inconsistent results. Turna et al. (2019) was the sole study from Canada (British Columbia) and found that 64.9% of patients reported a rating of 4 out of 5 (very effective) in alleviating their depression symptoms [16]. Despite this, the study also stated that this improvement was not clinically important and may have been confounded by those experiencing cannabis-withdrawal associated symptoms. Sexton et al. (2016) also reported a 86% reduction in depression-related symptoms as a result of cannabis use [4]. Conversely, Bahorik et al. (2017) reported that cannabis use worsened depression and the majority of patients had less improvements in depression symptoms [19]. Likewise, the two studies by Feingold et al. (2017) [17] and (2018) [18] reported that depression levels were markedly higher in medical cannabis users– but this study only compared the results to opioid users, and not the general population. The majority of the remaining clinical studies [10, 13, 21, 25] utilized other validation tools to measure depression outcomes and grouped depression under a general category of other mental health outcomes. Despite the mixed results, there was common observation amongst the studies that any initial increases in depression levels may not necessarily have been as a direct result of medical cannabis use [23].

The present study provides an important bridge for some of these knowledge gaps regarding cannabis and mental health outcomes. Indeed, depression is a major reason cited [16] for seeking medical cannabis authorization, hence the study provides important insight to potential safety and benefits regarding current and prospective users of medical cannabis. With the recent legalization of nonmedical cannabis in Canada, it is reassuring to observe that the majority of patients in this study did not experience a worsening of depression symptoms. These findings help contribute to the knowledge base on current and potential population health impacts of cannabis use, while at the same time, providing future direction to the field of mental health around cannabis research.

This analysis is currently the largest Canadian population-based study of medical cannabis use that we are aware of, but it is not without limitations. First, it is an observational study and thus, potential spectrum bias has to be considered as our cohort of patients are those who individually sought medical cannabis for treatment. Second, even though depression screening via the PHQ-9 is considered best practice for all clinicians, follow-up scores were not available for a large proportion of our cohort. Third, likewise to most pharmacoepidemiological studies, there is no absolute method for determining whether the medical cannabis authorized was consumed as prescribed, and if patients elected to use alternative treatments for depression. Fourth, comparative analysis with the outcomes from other studies remain challenging as previous studies predominantly do not utilize the PHQ-9 questionnaire to measure depression-related outcomes. Finally, our study is limited by the lack of clinical details: frequency, strain, quantity, and onset/age of depression symptoms.

## Conclusions

In all, we found no evidence of a therapeutic benefit associated to authorizing medical cannabis for patients seeking help with depression, depression-like conditions, disorders and related symptoms. Currently, there is very low evidence on medical cannabis and its effects on depression outcomes in both the short- and long-term. Future studies should focus on the parallel use of screening questionnaires to control for PHQ-9 sensitivity, ensure adherence to medical cannabis authorization, and whether the efficacy of cannabis to manage depression is comparable to first-line antidepressants. Lastly, future studies can further explore potential factors that may have attributed to the improvement in scores pre- and post- 3-6 month time period. Our findings contribute new evidence for clinicians on a large group of patients regarding the potential impact of medical cannabis for depression-related symptoms.

## Supplementary information

**Additional file 1: Table S1.** Keywords used to code the reason for seeking medical cannabis, method of cannabis use, and antidepressant usage.

**Additional file 2: Table S2.** Changes in PHQ-9 from initial to final follow-up for all patients with follow-up PHQ-9 scores and based on initial depression categorization (*n* = 5103).

**Additional file 3: Table S3.** Multiple Linear Regression Results.

## Data Availability

The dissemination of data results to study participants and or patient organizations in this research project is not possible/applicable. The data from the study will not be shared as only the researchers authorized by Ontario’s Institute for Clinical Evaluative Sciences (ICES) can have access to the data as per their policies.

## Abbreviations

PHQ-9: Patient Health Questionnaire
GAD-7: Generalized Anxiety Disorder 7-item scale
CAGE-AID: CAGE Questionnaire Adapted to Include Drugs
SSRI: Selective serotonin reuptake inhibitors
SNRI: Serotonin-norepinephrine reuptake inhibitors
TCA: Tricyclic antidepressants
NDRI: Norepinephrine–dopamine reuptake inhibitors
NaSSA: Noradnergic and specific serotonergic antidepressants
MAOI: Monoamine oxidase inhibitors
