# Supplementary material for: Changes in patient health questionnaire (PHQ-9) scores in adults with medical authorization for cannabis
Source: BMC Public Health. 2020 Jun 23;20:987. doi: 10.1186/s12889-020-09089-3 (PMC7310462; doi:10.1186/s12889-020-09089-3)
Supplement: Supplementary file 1 — Additional file 1: Table S1. Keywords used to code the reason for seeking medical cannabis, method of cannabis use, and antidepressant usage. [file 12889_2020_9089_MOESM1_ESM.pdf]

Supplemental Table 1. Keywords used to code the reason for seeking medical cannabis, method of cannabis use, and antidepressant usage

Categories	Keywords
Pain	Ache, carpal tunnel, carpel tunnel, cbp, ddd, degenerative disc, disc herniation, endometriosis, fibro, fracture, headache, herniated dis, injury, lbp, migrane, migraine, mva, mvc, myalgia, neuralgia, neuropathic, pain, radiculopa, sciatica, scoliosis, spasm, spina bif, spinal stenosis, spondylo, spondylitis, strain, tendin, tendon
Mental Health	Any keywords from the mental health subcategories as well as, add, adhd, ocd, schizo, stress
Anxiety	Anxiety, gad
Depression	Depression, depressive, mdd
Post-traumatic Stress Disorder	Post traumatic stress disorder, ptsd
Bipolar	Bipolar, bi-polar
Panic Disorder	Panic
Mood Disorder	Mood
Autoimmune	Ankylosing spond, ankylosis spond, arthral, arthrit, ibs, irritable bowel, lupus, ms, multiple sclerosis, oa, osteoarthrit, ra, rheumatoid, sjogren
Cancer	ca , cancer, carcinoma, glioblastoma, leukemia, lymphoma, malignant, melanoma, metastatic, myeloma, sarcoma, tumor, tumour
Sleep problems	Fatigue, insomnia, sleep, apnea
Neurological	Als, cerebral palsy, epilepsy, neuropathy, Parkinson, restless leg, seizure, tremor
Gastrointestinal	Chrohn, chron, colitis, crohn, ibs, irritable bowel
Other	Appetite, Asperger, autism, autistic, concussion, copd, diabet, glaucoma, huntington, nausea, osteop
Uncategorized	Did not contain any of the above keywords
Smoke	Smoke, smoking, bong, joint, pipe, dry, dried, bud
Vape	Vap, vaping, vapor, vapour, vaip
Ingest	Oil, drop, tab, pill, gel, caps, tinct, elix, edib, ediable, mouth, oral, subl, s/l, lingual, tongue, ingest, eat, bak, cook, food, drink, water, coffee, juice, tea, butter
Topical	Topica, cream
SSRI	sertraline, zoloft, fluoxetine, prozac, sarafem, citalopram, celexa, escitalopram, lexapro, cipralex, paroxetine, paxil, pexeva, brisdelle, fluvoxamine, luvox
SNRI	desvenlafaxine, pristiq, khedezla, duloxetine, cymbalta, irenka, levomilnacipran, fetzima, venlafaxine, effexor
TCA	amitriptyline, elavil, imipramine, tofranil, desipramine, norpramin, nortriptyline, aventyl, trimipramine, surmontil, clomipramine, anafranil
Other Antidepressants	bupropion, wellbutrin, zyban, mirtazapine, remeron, phenelzine, nardil, tranylcypromine, parnate
