# Supplementary material for: Changes in patient health questionnaire (PHQ-9) scores in adults with medical authorization for cannabis
Source: BMC Public Health. 2020 Jun 23;20:987. doi: 10.1186/s12889-020-09089-3 (PMC7310462; doi:10.1186/s12889-020-09089-3)
Supplement: Supplementary file 2 — Additional file 2: Table S2. Changes in PHQ-9 from initial to final follow-up for all patients with follow-up PHQ-9 scores and based on initial depression categorization (n = 5103). [file 12889_2020_9089_MOESM2_ESM.pdf]

Supplemental Table 2. Changes in PHQ-9 from initial to final follow-up for all patients with follow-up PHQ-9 scores and based on initial depression categorization (n=5103)

Change in PHQ-9 Score	N (%)					
	All Patients (n=5103)	Severe (n=648)	Moderately Severe (n=833)	Moderate (n=1069)	Mild (n=1372)	None (n=1181)
**Decrease**	172 (3.3)	33 (5.1)	41 (4.9)	59 (5.5)	39 (2.8)	-
-24	2 (0.0)	2 (0.3)	-	-	-	-
-22	1 (0.0)	1 (0.2)	-	-	-	-
-21	1 (0.0)	1 (0.2)	-	-	-	-
-20	2 (0.0)	2 (0.3)	-	-	-	-
-19	2 (0.0)	1 (0.2)	1 (0.1)	-	-	-
-18	3 (0.1)	2 (0.3)	1 (0.1)	-	-	-
-17	3 (0.1)	1 (0.2)	2 (0.2)	-	-	-
-15	4 (0.1)	2 (0.3)	2 (0.2)	-	-	-
-14	4 (0.1)	1 (0.2)	2 (0.2)	1 (0.1)	-	-
-13	4 (0.1)	1 (0.2)	2 (0.2)	1 (0.1)	-	-
-12	5 (0.1)	1 (0.2)	2 (0.2)	2 (0.2)	-	-
-11	14 (0.3)	2 (0.3)	2 (0.2)	10 (0.9)	-	-
-10	12 (0.2)	5 (0.8)	5 (0.6)	2 (0.2)	-	-
-9	16 (0.3)	1 (0.2)	7 (0.8)	6 (0.6)	2 (0.2)	-
-8	21 (0.4)	1 (0.2)	2 (0.2)	15 (1.4)	3 (0.2)	-
-7	19 (0.4)	2 (0.3)	2 (0.2)	8 (0.8)	7 (0.5)	-
-6	31 (0.6)	5 (0.8)	6 (0.7)	8 (0.8)	12 (0.9)	-
-5	28 (0.6)	2 (0.3)	5 (0.6)	6 (0.6)	15 (1.1)	-
**-4 to 4**	4855 (95.1)	615 (94.9)	787 (94.5)	998 (93.4)	1304 (95.0)	1151 (97.5)
5	20 (0.4)	-	1 (0.1)	4 (0.4)	6 (0.4)	9 (0.8)
6	10 (0.2)	-	1 (0.1)	2 (0.2)	5 (0.4)	2 (0.2)
7	12 (0.2)	-	1 (0.1)	3 (0.3)	4 (0.3)	4 (0.3)
8	9 (0.2)	-	-	1 (0.1)	6 (0.4)	2 (0.2)
9	4 (0.1)	-	1 (0.1)	1 (0.1)	1 (0.1)	1 (0.1)
10	5 (0.1)	-	1 (0.1)	-	2 (0.2)	2 (0.2)
11	6 (0.1)	-	-	-	2 (0.2)	4 (0.3)
12	4 (0.1)	-	-	1 (0.1)	1 (0.1)	2 (0.2)
13	1 (0.0)	-	-	-	-	1 (0.1)
14	2 (0.0)	-	-	-	2 (0.2)	-
17	1 (0.0)	-	-	-	-	1 (0.1)
20	1 (0.0)	-	-	-	-	1 (0.1)
27	1 (0.0)	-	-	-	-	1 (0.1)
**Increase**	76 (1.5)	-	5 (0.6)	12 (1.1)	29 (2.1)	30 (2.5)
