# Supplementary material for: Changes in patient health questionnaire (PHQ-9) scores in adults with medical authorization for cannabis
Source: BMC Public Health. 2020 Jun 23;20:987. doi: 10.1186/s12889-020-09089-3 (PMC7310462; doi:10.1186/s12889-020-09089-3)
Supplement: Supplementary file 3 — Additional file 3: Table S3. Multiple Linear Regression Results. [file 12889_2020_9089_MOESM3_ESM.pdf]

Supplemental Table 3. Multiple Linear Regression Results

Variable	β coefficient	Std. Error	t	P-value
Initial PHQ-9 Score	-0.051	0.005	-11.08	<0.001
Mental Health	-0.23	0.07	-3.06	0.017
Depression	0.34	0.10	3.41	0.001
Pain	0.19	0.08	2.40	0.017
SSRI Use	0.18	0.76	2.30	0.022
